# The phenotype of a *Plasmodium falciparum* phosphoeno/pyruvate carboxylase null mutant

**DOI:** 10.1186/1475-2875-9-S2-P49

**Published:** 2010-10-20

**Authors:** Janet Storm, Sylke Müller

**Affiliations:** 1 Institute for Infection, Immunity & Inflammation and Wellcome Trust Centre for Molecular Parasitology, College of Medical, Veterinary & Life Sciences, University of Glasgow, Glasgow, G1 2 8TA, UK

## 

CO_2_ is essential for the *in vitro* culture of intraerythrocytic *Plasmodium falciparum* and is thought to play a role in the parasite's carbon metabolism. CO_2_ fixation with phosphoeno/pyruvate is catalysed by phosphoeno/pyruvate carboxylase (PEPC) and supplies the cytosol with oxaloacetate (OAA), a process well defined in plants but absent from the mammalian host. In mammalian cells, the generation of OAA in the mitochondrion is achieved through pyruvate carboxylase, which is one of the most important anaplerotic reactions. The absence of a gene encoding pyruvate carboxylase in *Plasmodium* led to the hypothesis that cytosolic PEPC indirectly replaces this function by generating OAA, which is subsequently reduced to malate by a cytosolic, NADH-dependent malate dehydrogenase. Malate is then translocated into the mitochondrion and converted to OAA by malate:quinone oxidoreductase, which reduces the quinone pool of the electron transport chain. Another route cytosolic OAA can take is the conversion into aspartate by aspartate aminotransferase, which can be used for pyrimidine biosynthesis.

To assess whether *P. falciparum* PEPC is essential for parasite survival a reverse genetics approach was used. We were unable to disrupt the PEPC gene; however, when the cell culture medium was supplemented with millimolar concentrations of malate, a *PEPC* null mutant was generated. Interestingly, the null mutant was still viable without malate supplementation, but parasite growth was negatively affected. Supplementing the medium with excess malate or aspartate reversed the growth defect, but glutamine did not, suggesting that OAA forms a metabolic node in the cytosol orchestrating crucial metabolic reactions. Visual inspection of the parasites demonstrated a defect in trophozoite development implying that PEPC activity is important for the progression through intraerythocytic development.

